# Intraductal oncocytic papillary neoplasm arising in Peutz-Jeghers Syndrome bile duct: a unique case report

**DOI:** 10.1186/s13000-022-01275-8

**Published:** 2022-12-28

**Authors:** Qingyue Liu, Zhiyu Wang, Chaoran Yu, Jianping Zhu, Chengli Liu, Xiangsheng Li, Li Ren, Teng Li

**Affiliations:** 1 Department of Pathology, Air Force Medical Center, PLA, Beijing, People’s Republic of China; 2 China Medical University, Shenyang, People’s Republic of China; 3 Department of Hepatobiliary Surgery, Air Force Medical Center, PLA, Beijing, People’s Republic of China; 4 Department of Radiology, Air Force Medical Center, PLA, Beijing, People’s Republic of China

**Keywords:** Peutz-Jeghers syndrome, Intraductal oncocytic papillary neoplasm, *GNAS*, *STK11*, *EGFR*

## Abstract

**Background:**

Peutz-Jeghers syndrome (PJS) is a rare, autosomal dominant disorder caused by germline mutations of *STK11/LKB1*, with an increased risk of tumors at multiple sites. Intraductal oncocytic papillary neoplasm (IOPN) is a unique subtype of intraductal papillary neoplasm of the bile duct (IPNB) defined by a premalignant neoplasm with intraductal papillary or villous growth of biliary-type epithelium. IOPN has a distinct mutation profile compared with both IPNB and intraductal papillary mucinous neoplasm (IPMN).

**Case presentation:**

We herein describe the case of a 44-year-old woman who presented as polyps in the intestinal lumen of sigmoid colon and a 3.1 × 2.1 cm mass in the left lobe of liver. Gross feature revealed a cystic papillary mass and the neoplasm had a clear boundary with the surrounding liver tissue. Histology revealed complex papillary structures, a small amount of fine fibrovascular cores and immunohistochemistry showed extensive positive for MUC5AC, MUC6, CD117. Therefore, histological and immunohistochemical examination of the liver tumor suggested the diagnosis of IOPN. Next-generation sequencing (NGS) revealed other than *STK11* germline mutation, the tumor also harbors *GNAS* somatic mutation at codon 478 and *EGFR* amplification.

**Conclusion:**

To our knowledge, this is the first report of IOPN arising in PJS. This case enlarges the spectrum of PJS related tumors and genetic rearrangements in IOPN.

## Background

Peutz-Jeghers syndrome (PJS) is an autosomal dominant hereditary disease characterized by hamartomatous polyps of the gastrointestinal tract and mucocutaneous melanin deposits [1]. PJS is commonly caused by germline mutations in the tumor suppressor gene *LKB1/STK11* on chromosome 19 [2]. Previous studies have reported that patients with PJS had a significantly increased risk of gastrointestinal and extra-intestinal malignancies [1, 2]. Common extra-intestinal malignancies are breast cancer, ovarian cancer, cervical cancer, pancreatic cancer. Among them, the case of intraductal papillary mucinous neoplasm (IPMN) arising in Peutz-Jeghers syndrome is extremely rare [3]. Furthermore, intraductal papillary neoplasm of the bile duct (IPNB) with PJS has not been reported before. IPNB is an epithelial neoplasm with a tendency to progress into invasive cholangiocarcinoma and considered the biliary counterpart of IPMN [4]. Histologically, IPNB can be divided into four subtypes: intestinal, gastric, pancreatobiliary and oncocytic [4]. The oncocytic subtype of IPNB is also known as Intraductal oncocytic papillary neoplasm (IOPN) [5].

Previous studies have discovered genetic mutations in IPNB and IPMN through next-generation sequencing (NGS) method, such as *GNAS, KRAS, TP53, STK11, CTNNB1, RNF43, APC, SMAD4, EGFR*, etc. [4]. Recently, however, few reports suggest a distinct mutation profile of IOPN compared with IPNB and IPMN [5]. For example, *GANS* mutations are frequently found in both IPNB and IPMN [5]. While only one literature described IOPN could also harbor *GNAS* mutation [6]. As for *EGFR* mutations or amplifications, which are already uncommon in IPNB and IPMN, has not yet been reported in IOPN [7]. On the contrary, *STK11* mutations are more frequently seen in IOPN than IPNB and IPMN [8].

Here we report an extraordinarily rare case of IOPN arising in PJS, harboring *GNAS s*omatic mutation at codon 478, *EGFR* amplification and *STK11* germline mutation. To the best of our knowledge, this is the first case to report in literature. Therefore, the case report increases the spectrum of PJS related tumors and provides new genetic arrangements in IOPN.

## Case presentation

### Clinical history

**Fig. 1:**
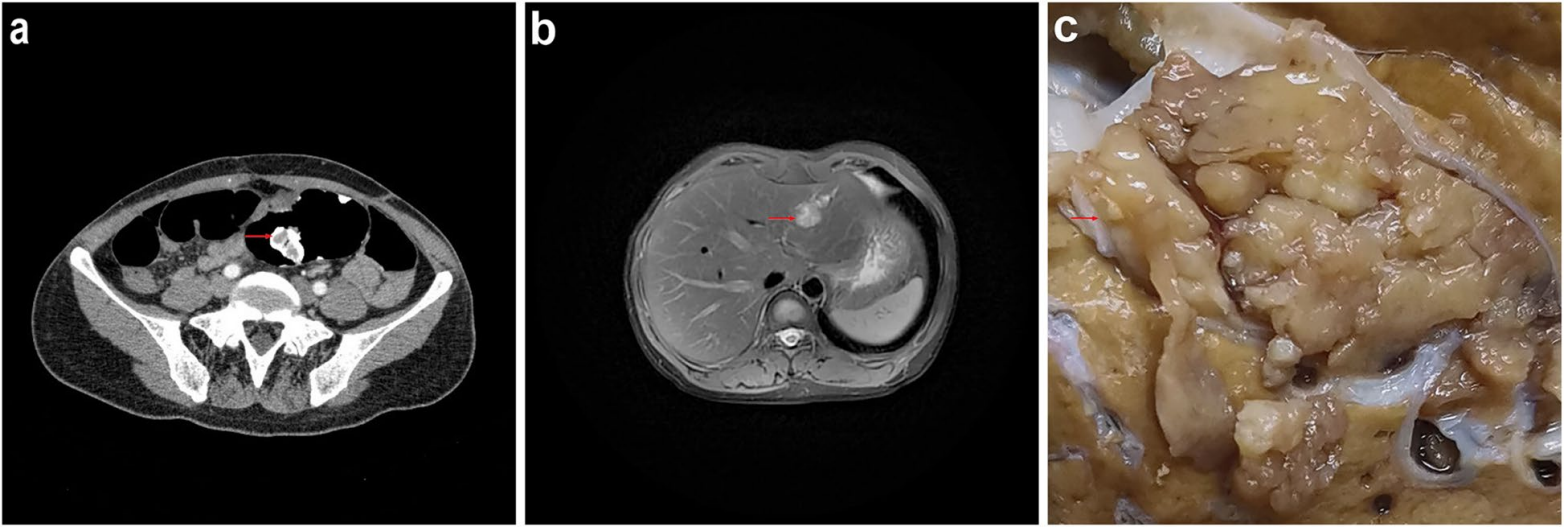
Pelvic CT and Abdominal MRI from the case and gross features of the neoplasm. **a** Pelvic CT showed nodular soft tissue shadow in the intestinal lumen of sigmoid colon, surrounded by high-density contrast agent. Red arrow indicates designate polyps. **b** Abdominal MRI showed a round low-density mass in the left lobe of liver with a clear border. Red arrow indicates designate IOPN. **c** Macroscopically, the neoplasm had a clear boundary with the surrounding liver tissue. Red arrow indicates designate IOPN

A 44-year-old Chinese woman was admitted to our hospital due to liver occupation during physical examination. Her suspected identical twin was a patient with PJS and died of colon cancer. Her other relatives do not suffer from PJS. And the patient had more than 30 years with pigmentation on the lip and intermittent abdominal pain for more than 10 years. Pelvic computed tomography (CT) scans revealed nodular soft tissue tumor in the intestinal lumen of sigmoid colon, about 2.3 × 1.0 cm in size, surrounded by high-density contrast agent, suggesting polyps (Fig. 1a). Abdominal color ultrasound showed the high echo area in the left lobe of liver with clear boundary and regular shape. Abdominal CT scans revealed a round low-density mass in the left lobe of liver with a size of 3.1 × 2.1 cm and a clear boundary. Abdominal magnetic resonance imaging (MRI) indicated mild expansion of the distal bile duct of the lesion in addition to the above mass (Fig. 1b). Abdominal color ultrasound, CT and MRI prompted suspicion of primary liver tumor.

### Pathological findings of resected specimens

**Fig. 2:**
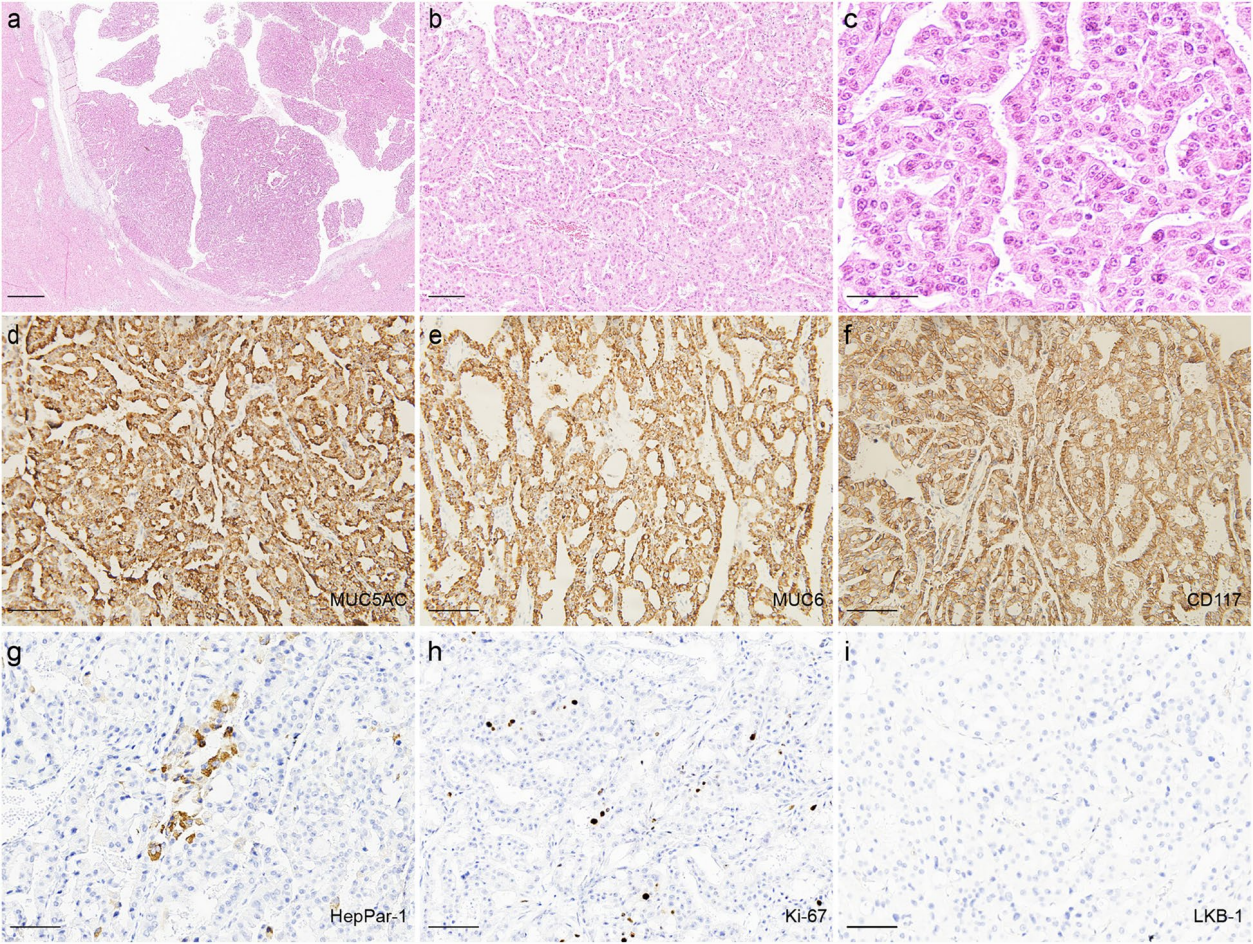
Histological and Immunohistochemical features of the neoplasm. **a** The bile duct wall and surrounding liver tissue were not invaded. (Scale bar = 1000 μm). **b** The neoplasm shows complex papillary structures, fine fibrovascular stalks. (Scale bar = 100 μm). **c** The tumor cells were abundant eosinophilic granular cytoplasm, round or oval nuclei, clear nucleoli. (Scale bar = 50 μm). **d-h** Tumor cells were positive for MUC5AC (**d**), MUC6 (**e**), CD117 (**f**), HepPar-1 (**g**) and the Ki-67 (**h**) index is relatively low. (All scale bars = 100 μm). **i** LKB1 staining was negative in the tumor cells. (Scale bar = 100 μm)

Grossly, a cystic papillary mass was seen in the hepatic bile duct, and the mass size was 2.1 × 2.8 × 1.7 cm. The distal hepatic bile duct was dilated. Macroscopically, the neoplasm had a clear boundary with the surrounding liver tissue, and did not invade the bile duct wall and surrounding liver tissue (Fig. 1c). Histology showed the mass was located in the hepatic bile duct. The bile duct wall and surrounding liver tissue were not invaded (Fig. 2a). The mass presented as complex papillary structures, a small amount of fine fibrovascular cores lined by 2 to 5 layers of cuboidal to columnar cells (Fig. 2b). And a small amount of mucus could be seen in the lumen. The neoplastic cells were abundant eosinophilic granular cytoplasm, with round or oval nuclei, clear nucleolus and the mitotic figure is rarely seen (Fig. 2c).

Immunohistochemical examination showed that tumor cells showed diffusely and intensely positive for MUC5AC, MUC6, CD117 (Fig. 2d–f), focal and patchy positivity of HepPar-1(Fig. 2g), multifocal positivity of S100P, MUC1 and negative for LKB1(Fig. 2h), CK20, MUC2, CDX-2. The Ki-67 proliferating index was 8% (Fig. 2i). Conclusively, our diagnosis of the hepatic lesion in this patient was “IOPN arising in PJS bile duct.”

### Molecular findings of resected specimens

**Fig. 3:**
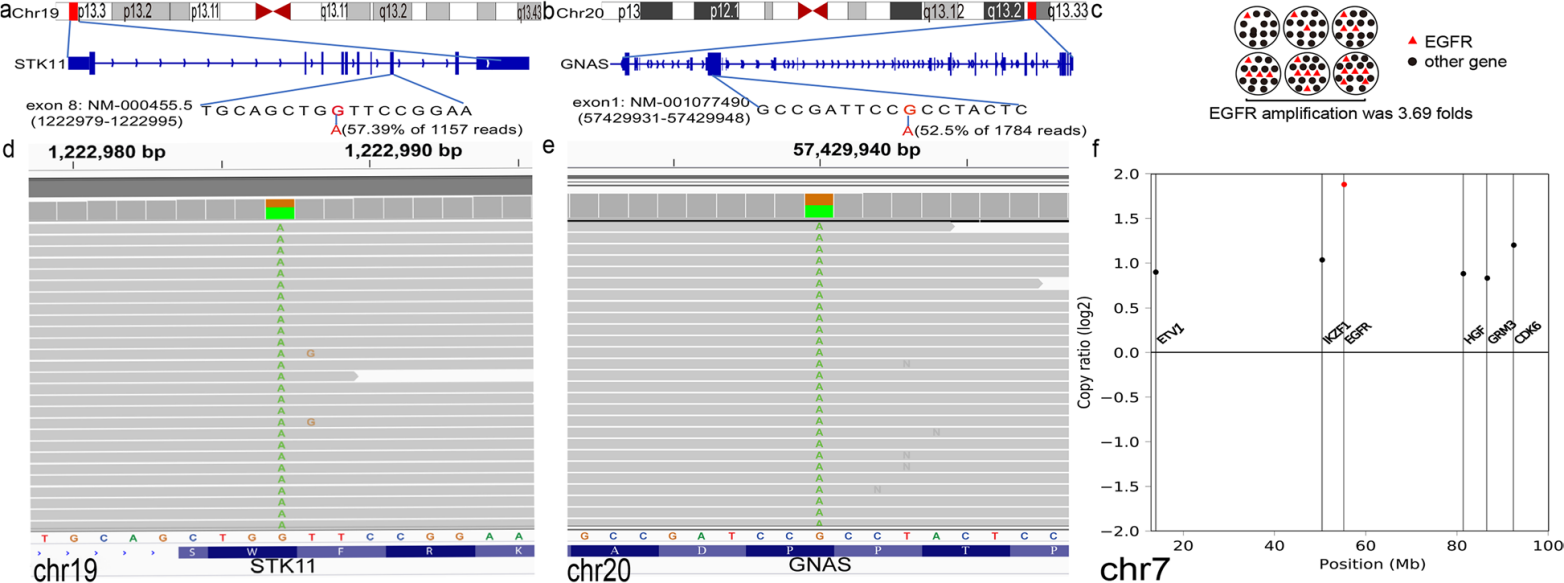
Molecular profile of the IOPN described in this case. **a-c** Schematic illustration of *STK11* (**a**) mutation, *GNAS* (**b**) mutation and *EGFR* (**c**) amplification. **d** The sequencing result showed *STK11* c.924G > A (p.W308*), exon 8 in 57.39% of 1157 reads mutation. **e** The sequencing result showed *GNAS* c.1433G > A (p.R478H), exon 1 in 52.5% of 1784 reads mutation. **f** The sequencing result showed *EGFR* amplification was 3.69 folds. (Data presented as log2 fold change at the FDR < 0.05)

Mutations included germline *STK11* c.924G > A (p.W308*), exon 8 in 57.39% of 1157 reads, *GNAS* c.1433G > A (p.R478H), exon 1 in 52.5% of 1784 reads, and *EGFR* amplification with 3.69 folds in the patient (Fig. 3a-c) (Results of complete NGS were summarized in Table 1) were detected by NGS through TruSight Oncology 500 from Illumina.

**Table 1:** Results of NGS in the patient

Mutation	Reads
STK11 c.924G > A (p.W308^*^), exon 8	57.39%/1157
GNAS c.1433G > A (p.R478H), exon 1	52.50%/1782
FOXP1 c.174-176del (p. Q59del), exon 6	1.80%/3219
SF3B1 c.1849 A > G (p.I617V), exon 14	1.90%/1263
TRAF7 c.759-2 A > G, exon 10	3.08%/843
TRAF7 c.805 A > G (p.1269 V), exon 10	3.90%/950
TRAF7 c.815 A > C (p.Q272P), exon 10	4.53%/1016
EGFR amplification with 3.69	NA
NA indicates not available	

### Treatments and outcome

The patient underwent hepatic left lateral lobectomy due to liver neoplasm. After 18 months of follow-up by CT and MRI surveillance, no evidence of tumor recurrence or progression was observed.

## Discussion and conclusion

IOPN is an epithelial neoplasm derived from the pancreatobiliary system. In contrast to the other types of IPNB, IOPN is composed of complex, arborizing papillae lined by one to several stratified layers of cuboidal to columnar cells, and the cells are abundant eosinophilic granular cytoplasm, hyperchromatic, round, large, and fairly uniform nuclei [9]. Symptoms of IOPN are not specific. IOPN is frequently presented with intermittent biliary obstruction, recurrent pyogenic cholangitis, jaundice, intermittent abdominal pain, nausea, and weight loss [9]. Molecular studies of IOPN are scant [5, 6, 8, 10–18]. Here, we have summarized previous publications of molecular alterations of IOPN (both pancreatic and biliary) in Table 2. As we can see from Table 2, IOPN rarely harbors *GNAS* mutations, which are frequently found in both IPMN and IPNB. Other than gene mutations, recent publications have discovered recurrent rearrangements in *PRKACA* and *PRKACB* in IOPN [5, 17, 18]. These fusions result in increased *PRKACA* or *PRKACB* expression and, consequently, an increase in Protein Kinase A (PKA) activity [5, 17, 18].

**Table 2:** Review of reported molecular alterations of IOPN

References	Upregulated gene	Fusion	Loss of heterozygosity	Amplification	Gene mutation	Origin
Xiao et al. [10]					KRAS, BRAF	Pancreas
Mohri et al. [11]					KRAS	Pancreas
Schlitter et al. [12]			P16			Bile Duct
Amato et al. [6]					GNAS, KRAS	Pancreas
Basturk et al. [13]					ARHGAP26, ASXL1, EPHA8, ERBB4	Pancreas
Singhi et al. [5]		ATP1B1-PRKACA, ATP1B1-PRKACB, DNAJB1-PRKACA				Pancreas, Bile Duct
Vyas et al. [18]		DNAJB1-PRKACA, PRKACA-ATP1B1				Pancreas, Bile Duct
Aoki et al. [14]					TP53, KRAS, PBRM1, ELF3, NF1, BAP1, EPHA6, ERBB3, KMT2D, CDKN2A	Bile Duct
Nakahodo et al. [15]	Follistatin (FST)					Pancreas, Bile Duct
Chang et al. [16]					KRAS	Pancreas
Omori et al. [8]			P16		STK11, KRAS, P53, SMAD4	Pancreas
Maimaitiaili et al. [17]		ATP1B1-PRKACA, DNAJB1-PRKACA ATP1B1-PRKACB			KRAS	Pancreas, Bile Duct
This case				EGFR	STK11, GNAS	Bile Duct

NGS revealed the patient in our study mainly had *STK11* mutation, *GNAS* mutation and *EGFR* amplification. *STK11* is a tumor suppressor gene, which acts to restrict cell growth via mTOR inactivation and induction of other AMP(adenosine monophosphate)-activated protein kinase (AMPK)-related kinases [19]. *GNAS* mutation leads to constitutive activation of the cyclic adenosine monophosphate (cAMP)-PKA signaling pathway [20]. This causes upregulation of epidermal growth factor receptor (EGFR) [21]. EGFR is a 170-kDa monomeric glycoprotein [22].When *EGFR* is amplified, it stimulates cell proliferation via EGFR-mechanistic target of rapamycin (mTOR) Pathway [23]. On the one hand, *STK11* defection promotes cell growth by activating mTOR pathway. On the other hand, *GNAS* mutation and *EGFR* amplification promote cell growth by activation of PKA, overexpression of EGFR and stimulation of mTOR. Therefore, we propose that *STK11* mutation, *GNAS* mutation and *EGFR* amplification together promote tumorigenesis of IOPN. Both *GNAS* mutations and *PRKACA* and *PRKACB* fusions result in an increase in PKA activity [5, 17, 18]. This suggest that in our case, the function of *GNAS* mutation might be “mimicking” *PRKACA* and *PRKACB* fusions in the development of IOPN.

In this case, the tumor showed *GNAS* gene mutation at codon 478 (exon 1, c.1433G > A). While previous research only reported that IPNB and IPMN have *GNAS* mutations at codon 201 [4, 24]. This novel mutation locus adds to the spectrum of genetic mutations associated to IPNB. Simultaneously, it suggests that the *GNAS* mutation locus in IOPN may be different from other subtypes of IPNB and IPMN.

In conclusion, we have described for the first time a rare case of IOPN arising in PJS. Current surveillance programs for PJS are enforcement both for prevention of gastrointestinal complications and for early detection of relevant malignancies [2]. IOPN is not part of the known spectrum of PJS, thus we suggest that the early screening of pancreatobiliary system could also be important in the prevention of PJS related malignancies.

## Data Availability

The datasets used and/or analyzed during the current study are available from the corresponding author on reasonable request.

## Abbreviations

PJS: Peutz-Jeghers syndrome
IOPN: intraductal oncocytic papillary neoplasm
IPNB: intraductal papillary neoplasm of the bile duct
IPMN: intraductal papillary mucinous neoplasm
NGS: next-generation sequencing
CT: computed tomography
MRI: magnetic resonance imaging
FST: follistatin
PKA: protein kinase A, cAMP-dependent protein kinase
AMP: adenosine monophosphate
AMPK: AMP-activated protein kinase
cAMP: cyclic adenosine monophosphate
EGFR: epidermal growth factor receptor
mTOR: mechanistic target of rapamycin
